# The Effects of Apigenin on the Expression of Fas/FasL Apoptotic Pathway in Warm Liver Ischemia-Reperfusion Injury in Rats

**DOI:** 10.1155/2014/157216

**Published:** 2014-07-06

**Authors:** Evanthia G. Tsalkidou, Alexandra K. Tsaroucha, Ekaterini Chatzaki, Maria Lambropoulou, Fotini Papachristou, Gregory Trypsianis, Michael Pitiakoudis, Georgios Vaos, Constantinos Simopoulos

**Affiliations:** ^1^Second Department of Surgery, Medical School, Democritus University of Thrace, University Hospital of Alexandroupolis, 681 00 Alexandroupolis, Greece; ^2^Laboratory of Experimental Surgery and Surgical Research, Medical School, Democritus University of Thrace, University Hospital of Alexandroupolis, 681 00 Alexandroupolis, Greece; ^3^Department of Pharmacology, Medical School, Democritus University of Thrace, University Hospital of Alexandroupolis, 681 00 Alexandroupolis, Greece; ^4^Laboratory of Histology, Medical School, Democritus University of Thrace, University Hospital of Alexandroupolis, 681 00 Alexandroupolis, Greece; ^5^Laboratory of Medical Statistics, Medical School, Democritus University of Thrace, University Hospital of Alexandroupolis, 681 00 Alexandroupolis, Greece

## Abstract

*Background*. The aim of this experimental study was to investigate the role of apigenin in liver apoptosis, in an experimental model of hepatic ischemia-reperfusion in rats. *Materials and Methods*. Forty-eight Wistar rats (apigenin and control groups), 14 to 16 weeks old and weighing 220 to 350 g, were used. They were all subjected to hepatic ischemia by occlusion of the hepatic artery and portal vein for 45 minutes and reperfusion was followed for 60, 120, and 240 minutes. Apigenin was administrated intraperitoneally. Liver tissues were used for the detection of apoptosis by TUNEL assay and caspase 3 antibodies. Expression analysis of *Fas/FasL* genes was evaluated by real time PCR. *Results*. The expression analysis of *Fas* and *FasL* genes was increasing during reperfusion (significantly in the group of 240 minutes of reperfusion). It was in the same group that apigenin decreased Fas receptor levels and inhibited apoptosis as confirmed by TUNEL assay and caspase 3 antibodies. *Conclusions*. The effects of apigenin in the Fas/FasL mediated pathway of apoptosis, in the hepatic ischemia-reperfusion, seem to have a protective result on the hepatic cell.

## 1. Introduction

Ischemia-reperfusion injury is a phenomenon whereby cellular damage in a hypoxic organ is accentuated following the restoration of oxygen delivery. It affects oxygen-dependent cells of tissues and organs such as heart, brain, liver, kidney, and intestine [1–3]. In liver surgery, there are clinical situations where the ischemic periods can be particularly long, such as during the resection of large hepatic tumors, management of hepatic trauma of diverse origins, vascular reconstructions, and liver procurement for transplantation [4–6]. In the ischemic phase, anoxic injury of oxygen-dependent cells is clearly the predominant injury process. Once the blood flow and oxygen supply are reestablished, reperfusion enhances the injury caused by the ischemic period, aggravating the damage caused at the cellular level, as an injurious inflammatory response is also involved. This cell injury occurring in the reperfusion phase may either be a consequence of cellular alterations that were already initiated in the ischemic phase or may result from the inflammatory response [7]. The severity of cell injury can lead to either hepatocyte necrosis or apoptosis [8]. In order to protect the hepatic parenchyma from ischemia-reperfusion injury, research continues to focus on the elucidation of the exact mechanisms of apoptosis.

In the liver, the death receptors Fas, TNF receptor 1 (TNF-R1), and TRAIL receptor 1/2 (TRAIL-R1/2) are major mediators of the apoptotic pathway. Upon stimulation by their ligands, FasL, TNF-*α*, and TRAIL, respectively, the death receptors oligomerize and recruit different adaptor proteins which activate the initiator caspase 8. Fas is constitutively expressed by every cell type in the liver. Its ligand, FasL, is mainly expressed as a transmembrane protein on the cell surface of activated cytotoxic T lymphocytes. Kupffer cells also express FasL in response to engulfment of apoptotic bodies [9].

Independently of the apoptotic pathway, the first morphological changes appear at the cell membrane. In an interval of 1 to 3 hours, cell to cell adhesion decreases and either the cytosolic or mitochondrial proteins are altered resulting in nuclear changes. The ultimate determinant of apoptosis is an intranucleosomal DNA fragmentation [10]. As the biochemical hallmark of programmed cell death is initiated by the caspase cascade that ultimately leads to DNA fragmentation, both caspase activation and DNA fragmentation need to be demonstrated to establish if a cell has undergone the process of apoptosis [11].

The TUNEL assay is based on the specific binding of terminal deoxynucleotidyl transferase (TdT) to 3 = –OH ends of fragmented DNA. Following proteolytic treatment of histological sections, TdT incorporates X-dUTP (X-biotin, DIG, or fluorescein) at sites of DNA breaks. Termini modified nucleotides amplify the signal and allow the examination of labelled cells under microscopy, flow cytometry, or immunohistochemistry [10].

On the other hand, as caspases are essential effector molecules of apoptosis, assaying for cleaved caspases offers the detection of apoptosis in early stages. Monoclonal antibodies are available against all the caspases for immunohistochemistry, ELISA, and even some for flow cytometry (i.e., active caspase 3) [12].

Each method that is able to detect and verify apoptosis has advantages and disadvantages, and it is best to confirm apoptosis with multiple complementary techniques [12].

Inhibition of apoptosis seems to improve hepatocyte survival and prevent reperfusion injury. Several substances that modulate apoptotic processes have been used in order to protect the hepatic parenchyma. Apigenin, one of the most common flavonoids, known for its antioxidant and anti-inflammatory properties, has been studied for its possible protective action in ischemia-reperfusion injury [13–16]. The aim of this experimental model of ischemia-reperfusion in rats was to investigate the effects of apigenin on the expression of Fas/FasL apoptotic pathway.

## 2. Materials and Methods

### 2.1. Animals and Experimental Protocol

Forty-eight Wistar-type rats, aged between 14 and 16 weeks and weighing 220 to 350 g, were used. All experiments described were performed according to the European Union's guidelines for the ethical treatment of experimental animals. Rats were randomized in eight groups and anesthetized by using diethyl ether and sevoflurane. After laparotomy they were all subjected to hepatic ischemia by occlusion of the hepatic artery and portal vein for 45 minutes using atraumatic micro vascular clips. Apigenin (5 mg) was administrated intraperitoneally in solution of 0.3 mL NaCl 0.9% and 0.2 mL dimethyl-sulfoxide solvent (DMSO) [17]. A dose of 15 mg per kilo of body weight was administrated. Reperfusion was followed for 60, 120, and 240 minutes (three groups named AP60, AP120, and AP240, resp.). The same number of rats was used as control groups subjected only to ischemia and reperfusion for the respective times (C60, C120, and C240). In two more groups of animals, a sham operation of open-close laparotomy (group C) and a sham operation using solution of 0.3 mL NaCl 0.9% and 0.2 mL DMSO intraperitoneally (DMSO group) were performed. After completion of the reperfusion time, all animals were euthanized and liver tissues were used for the detection and expression analysis of* Fas* and* FasL* genes by real time PCR. The terminal deoxynucleotidyl transferase-dUTP nick end labelling, TUNEL assay, and the use of caspase 3 antibodies were also performed in all liver tissues, in order to confirm apoptosis.

### 2.2. Measuring the Expression of* Fas/FasL* Genes

Liver tissues were subjected to homogenization by using a solution of phenol-guanidine and chloroform, and the RNA samples were isolated. These isolated samples, after elaboration with deoxyribonuclease I, in order to remove the residues of DNA, were first reverse transcripted to cDNA with reverse transcriptase, and then the mRNA quantity of each sample was measured using real time polymerase chain reaction (RT-PCR). The whole process took place in a Light Cycler MX3005P using the SYBR MM QPCR Brilliant mix (Stratagene) as fluorescent reporter.

The expression of Fas/FasL was calculated by comparing the amplification plots with the method of MaxPro QPCR Software Version 3 (Stratagene). Analysis of relative gene expression data was performed according to the 2^−DDCT^ (Livac and Schmittgen 2001) using *β*-actin as an endogenous reference gene and cDNA from total rat brain extract as a control reference sample.

### 2.3. Detecting Apoptosis by Immunohistochemistry

Paraffin embedded tissue samples from liver tissue were available for all animals. Unstained slides were obtained for the detection of apoptosis using the monoclonal antibodies caspase 3 (Santa Cruz Biotechnology, Inc., USA) and TUNEL reaction mixture (Roche Applied Science, Germany). Tissue specimens were fixed in formalin and embedded in paraffin according to standard histological procedures. Four-micron sections (4 *μ*m) of representative blocks from each case were deparaffinized, rehydrated, and treated with 0.3% H_2_O_2_ for 5 min in methanol to prevent endogenous peroxidase activity and were immunostained by the peroxidase method (Envision System, DAKO, Carpinteria, CA, USA). Slides were then incubated for 60 minutes with the antibodies caspase 3 at a 1 : 200 dilution. Control slides were incubated for the same period with nonimmunized rabbit serum (negative control). A positive control was always run in the assay.

Slides for TUNEL method were incubated for 75 min with the TUNEL reaction mixture (Roche Applied Science, Germany) according to the manufacturer's protocol. Control slides were incubated for the same period with 50 *μ*L label solution (negative control).

Finally, bound antibody complexes were stained for 10 min with 0.05% diaminobenzidine. Sections then were briefly counterstained with Mayer's haematoxylin, mounted, and examined under a Nikon Eclipse 50i microscope. The expression of antibodies was cytoplasmic or nuclear.

The positive expression of caspase 3 and TUNEL was determined by counting the number of stained cells (cytoplasmic or nuclear localization). Sections with greater than 10% stained cells were considered positive (0: negative; 1: low; 2: moderate; and 3: high expression). The average labeling index of caspase 3 and TUNEL was assessed according to the proportion of positive cells, after scanning the entire section of the specimen. The results of expression were graded as negative (0) for <10% of stained cells, low (1) for >10% and <30% of cells stained, moderate (2) for >30% and <70% cells stained, and high (3) for >70% cells stained.

### 2.4. Statistical Analysis

Statistical analysis of data was performed using SPSS (Statistical Package for Social Sciences) v.14.0. All values were expressed as the mean ±1 for statistical error. The statistical evaluation of significantly different levels of Fas/FasL was obtained using the Student's *t*-test, while the equality of the means of the several groups was provided by analysis of variance (ANOVA). All statistics tests were subjected to a two-way analysis and *P* values <0.005 were considered statistically significant.

## 3. Results

Levels of Fas/FasL proteins in every sample were calculated by comparing the amplification plots as they resulted from the program of Real Time PCR MxPro.

**Figure 1:**
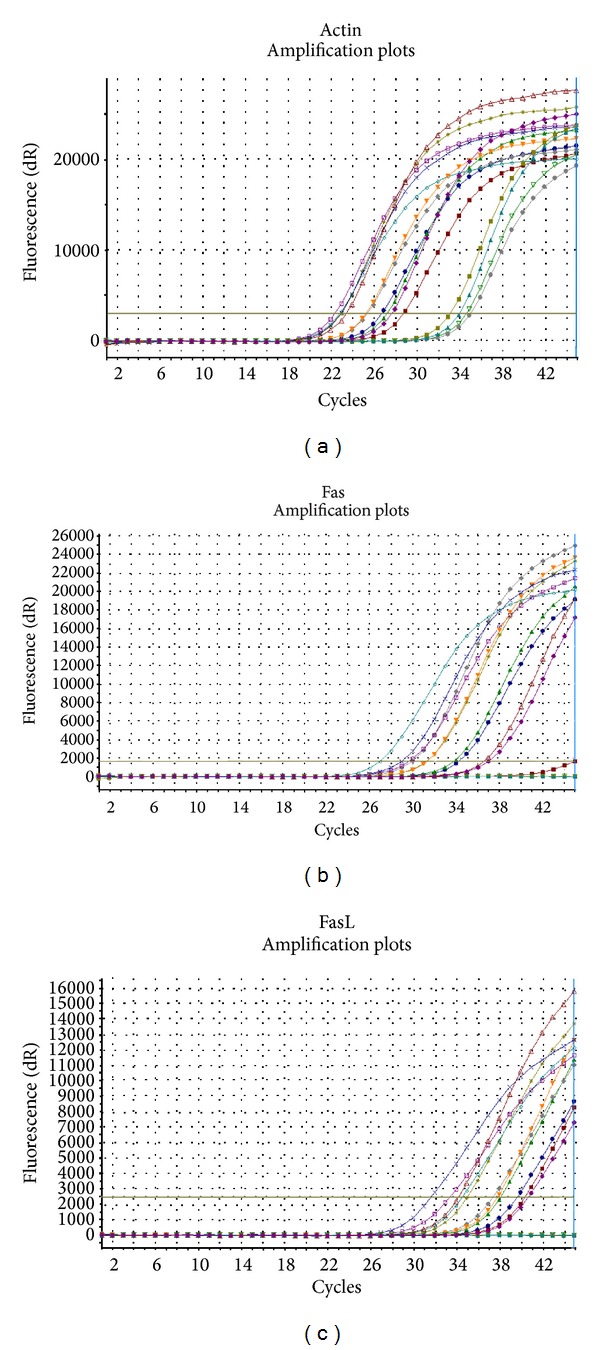
Amplification plots of (a) actin gene; (b)* Fas* gene; and (c)* FasL* gene.

Controlling the amplification plots, negative control samples, that is, samples without reverse transcriptase, were not multiplied confirming the reliability of the experiment (Figure 1).

**Figure 2:**
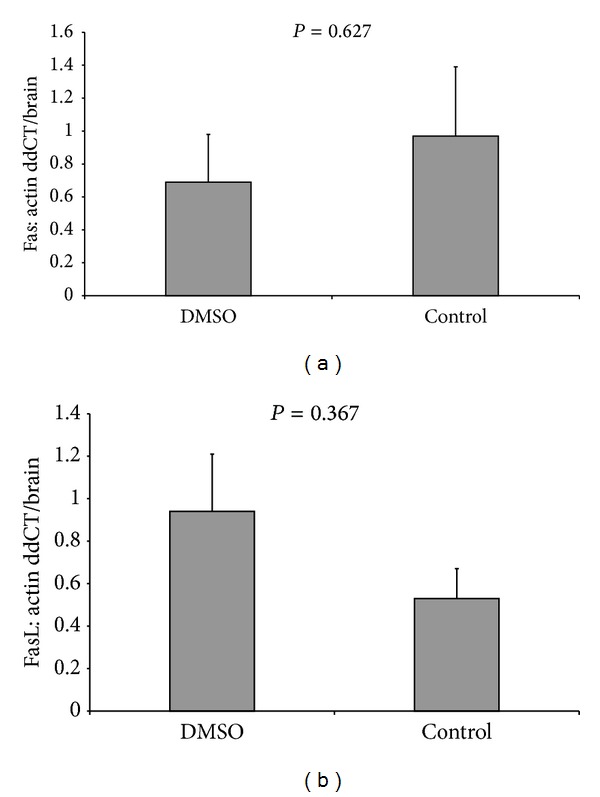
Levels of (a) Fas protein in group C and DMSO and (b) FasL protein in group C and DMSO.

In order to check the absence of solvent's toxicity, the comparison of Fas protein levels in group C (sham operated rats) and group DMSO (sham operated rats with use of solvent DMSO intraperitoneally) was performed first. The difference of Fas levels in these two groups was not significant (*P* = 0.627). The same result aroused for the FasL levels when they were compared in the same two groups (*P* = 0.367; Figure 2).

**Figure 3:**
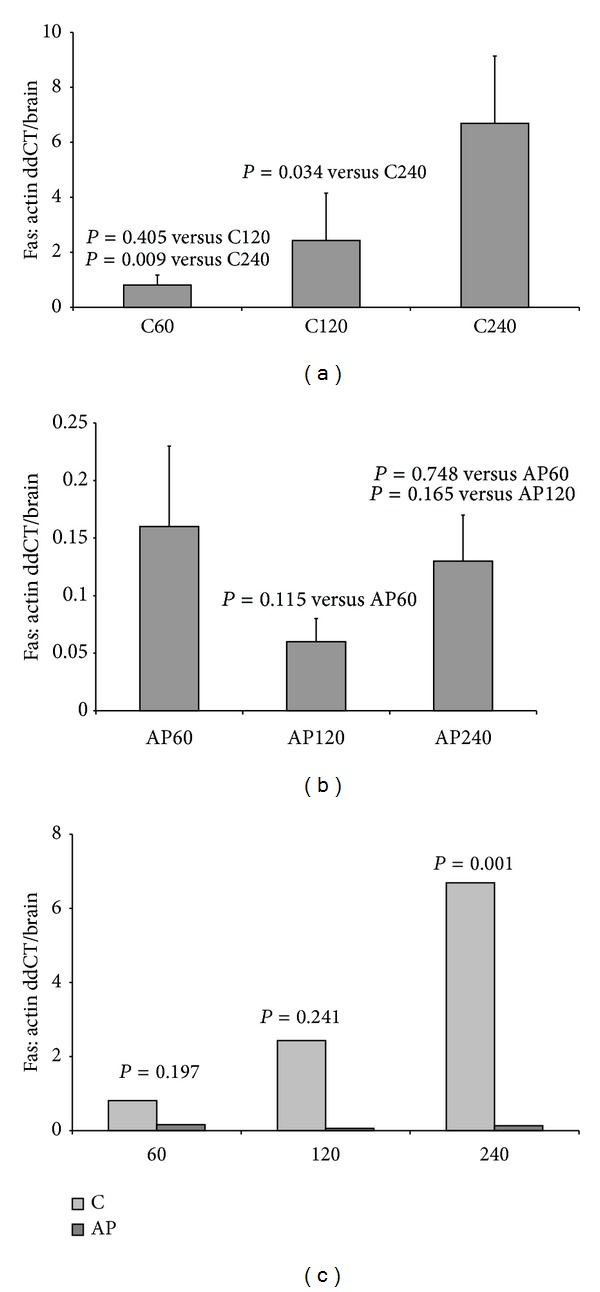
Levels of Fas protein in groups: (a) C60, C120, and C240; (b) AP60, AP120, and AP240; and (c) C60-AP60, C120-AP120, and C240-AP240.

Consecutively, the Fas protein levels were compared among the control groups C60, C120, and C240 (Figure 3(a)), among the groups where apigenin was used AP60, AP120, and AP240 (Figure 3(b)), and finally, among the groups subjected to the same time of reperfusion, with and without the use of apigenin, that is, C60 versus A60, C120 versus AP120, and C240 versus AP240 (Figure 3(c)).

As shown in Figure 3(a), Fas protein levels are increasing with the time of reperfusion and this increase in the C240 group was statistically significant compared to C120 (*P* = 0.034) and C60 groups (*P* = 0.09). In groups where apigenin was administrated after the removal of ischemia, a reduction of protein levels was marked 60 min after reperfusion, that is, in AP120 and AP240 groups compared to AP60 group, but the reduction was not significant (*P* = 0.115 for AP120 and *P* = 0.748 for AP240). At the end, as shown in Figure 3(c), the reduction of Fas protein levels was statistically significant concerning the group where apigenin was administrated and reperfusion lasted 240 minutes (*P* = 0.001). In the remaining groups, although a reduction of Fas levels was noticed, this was not significant (*P* = 0.197 for groups C60-AP60 and *P* = 0.0241 for groups C120-AP120).

**Figure 4:**
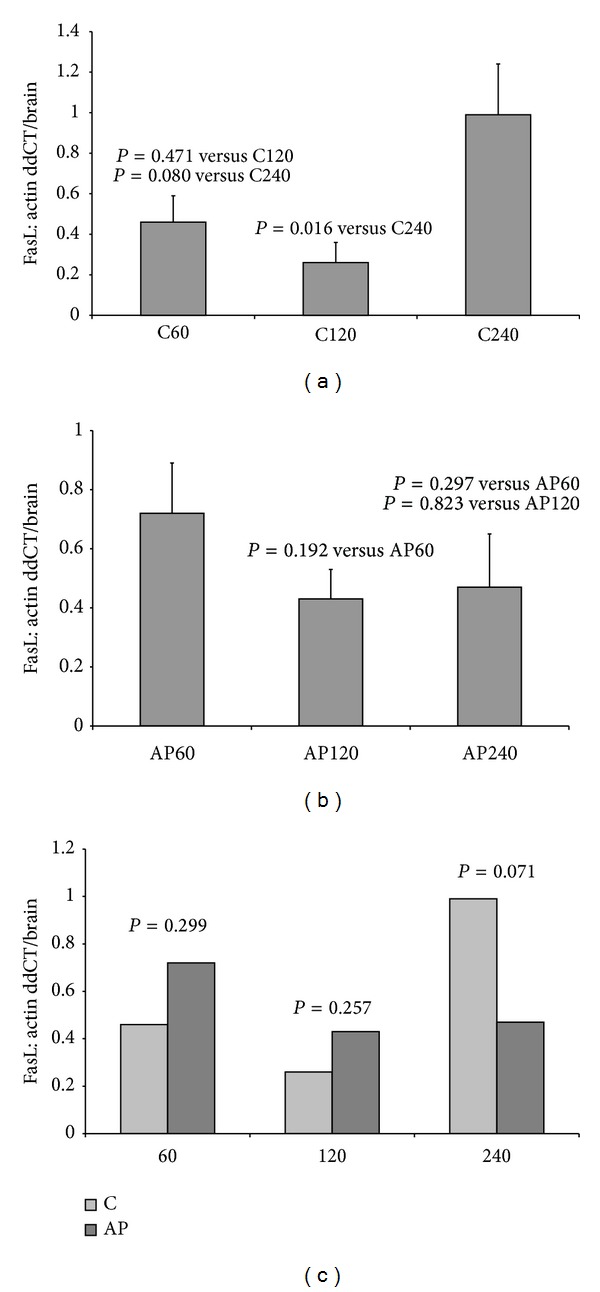
Levels of FasL protein in groups: (a) C60, C120, and C240; (b) AP60, AP120, and AP240; and (c) C60-AP60, C120-AP120, and C240-AP240.

Exactly the same procedure was followed regarding the FasL protein levels (Figure 4). In the control groups, the levels of FasL protein were increased significantly in C240 group when it was compared to C120. The same was not valid for the rest of the groups. In Figure 4(b), groups with apigenin administration are compared. Although a reduction of FasL levels was noticed 120 and 240 minutes after reperfusion, this reduction was not statistically significant. Completing the comparisons among C60-AP60, C120-AP120, and C240-AP240, a reduction of FasL levels, even not statistically significant, was observed only in the group of apigenin after 240 minutes of reperfusion (Figure 4(c)).

**Figure 5:**
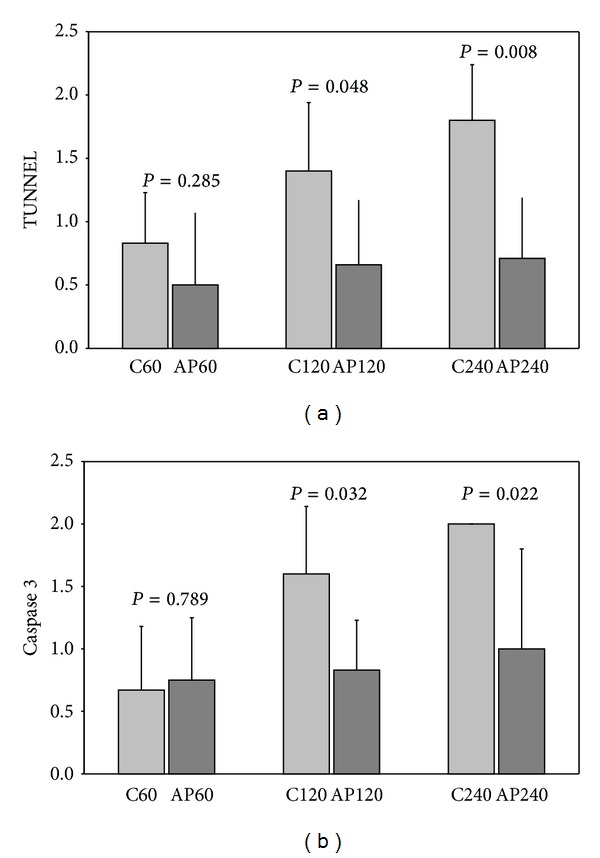
Apoptosis in groups: (a) C60-AP60, C120-AP120, and C240-AP240 by the TUNEL assay and (b) C60-AP60, C120-AP120, and C240-AP240 by caspase 3.

When the TUNEL assay was performed, statistically significant higher values in controls compared to the apigenin group were observed (*P* = 0.048 at 120 min and *P* = 0.008 at 240 min) (Figure 5(a)). Then, caspase 3 levels were evaluated, and similar results were produced, that is, lower levels of caspase 3 in all apigenin groups compared to the control groups. The difference was also found statistically significant in AP120 and AP240 groups (*P* = 0.032 at 120 min and *P* = 0.022 at 240 min; Figure 5(b)).

## 4. Discussion

A period of ischemia is required for a number of surgical procedures on the liver, especially when dealing with extensive hepatic trauma or resecting large intrahepatic lesions [18, 19]. On restoring the blood supply, the liver is subjected to a further impact, aggravating the injury already caused by ischemia. This is termed ischemia-reperfusion injury, and in the field of hepatic transplantation, it is responsible for graft function and even viability [20]. The pathophysiology of hepatic ischemia-reperfusion includes a number of mechanisms that contribute to various degrees in the overall injury [2]. Hepatic cell death occurs due to both necrosis and apoptosis [21]. These two modes of cell death seem to coexist in liver pathology as interdependent phenomena resulting from activation of shared pathways and signals [22]. In some cases, it is the type of stimuli and/or the degree of stimuli that determines if cells die by apoptosis or necrosis [23].

The mechanisms of apoptosis are highly complex, involving an energy dependent cascade of molecular events. These two are the main apoptotic pathways: the intrinsic or mitochondrial and the extrinsic or death receptor pathway [24]. Fas, a transmembrane protein that belongs to the tumor necrosis factor superfamily, is included in the death receptors of importance in liver. Hepatocytes, cholangiocytes, sinusoidal endothelial cells, stellate cells, and Kupffer cells all express Fas protein. Death receptor engagement by its corresponding ligand FasL causes receptor activation and the beginning of the apoptotic cascade [25].

The aim of this experimental study was to investigate the role of apigenin in the Fas/FasL mediated pathway of apoptosis, in an experimental model of hepatic ischemia-reperfusion in rats. The presence of apoptosis was confirmed by terminal deoxynucleotidyl transferase-dUTP nick end labelling (TUNEL) assay and by using caspase 3 antibodies.

Apigenin, known for its antioxidant, anti-inflammatory, and anticancer properties, was used intraperitoneally, since intraperitoneal administration obtains better absorption. Apigenin was dissolved in DMSO solvent and was administered after reperfusion. The security of the solvent was demonstrated by the comparison of Fas and FasL levels between the control groups C and the DMSO groups, where pure solvent was used. The difference was not statistically significant, so DMSO did not affect the results.

In this experimental study, the expression of the transmembrane protein Fas was increasing with reperfusion duration. This expression was statistically significant 240 minutes after reperfusion. The same result was observed on the expression of the Fas ligand protein. The degree of the reperfusion injury, that is, apoptotic death in hepatic parenchyma, started to be obvious two hours after reperfusion and the difference became significant four hours after reperfusion. This demonstrates an increase of apoptotic procedure, which confirms the aggravation of cell injury after reperfusion in cells subjected to ischemia.

Regarding the effect of apigenin on the expression of Fas receptor, a reduction of protein levels was noticed in all groups where the substance was administered, when control groups were compared to respective apigenin groups. The reduction was statistically significant in AP240 group, indicating a possible protective effect of apigenin in case of hepatic ischemia-reperfusion after the first two hours of blood restoration. A reduction was marked for the Fas ligand protein but it was not statistically significant. At the end, although the higher concentration of Fas and FasL protein was noticed in the AP60 group, compared to AP120 and AP240, the results were not statistically significant.

Even if Fas receptor is included in the major mediators of apoptotic pathways in liver, regulation of Fas/FasL levels alone does not determine apoptosis but could only represent an indirect proof of apoptosis inhibition. Apoptosis is directly determined in this experiment by TUNEL method and caspase 3 levels. Both methods confirmed the inhibiting effect of apigenin on apoptosis in groups of 120 and 240 minutes of reperfusion.

## 5. Conclusions

The effects of apigenin in Fas/FasL mediated pathway of apoptosis in the experimental model of hepatic-reperfusion in rats seem to be encouraging. Specifically the expression of* Fas* gene in hepatocytes was increased during reperfusion and especially four hours after. Exactly at the same time, apigenin reduced the concentration of Fas receptor. Further studies on apigenin effects in hepatic ischemia-reperfusion may prove to be of great importance in liver surgery.

## References

[1] Teoh NC, Farrell GC (2003). Hepatic ischemia reperfusion injury: pathogenic mechanisms and basis for hepatoprotection. *Journal of Gastroenterology and Hepatology*.

[2] Jaeschke H (2003). Molecular mechanisms of hepatic ischemia-reperfusion injury and preconditioning. *The American Journal of Physiology—Gastrointestinal and Liver Physiology*.

[3] Jaeschke H (1998). Mechanisms of reperfusion injury after warm ischemia of the liver. *Journal of Hepato-Biliary-Pancreatic Surgery*.

[4] He SQ, Zhang YH, Venugopal SK (2006). Delivery of antioxidative enzyme genes protects against ischemia/reperfusion-induced liver injury in mice. *Liver Transplantation*.

[5] Devasagayam TPA, Tilak JC, Boloor KK, Sane KS, Ghaskadbi SS, Lele RD (2004). Free radicals and antioxidants in human health: current status and future prospects. *Journal of Association of Physicians of India*.

[6] Halliwell B (1996). Antioxidants in human health and disease. *Annual Review of Nutrition*.

[7] Montalvo-Jave EE, Escalante-Tattersfield T, Ortega-Salgado JA, Piña E, Geller DA (2008). Factors in the Pathophysiology of the Liver Ischemia-Reperfusion Injury. *Journal of Surgical Research*.

[8] Kerr JFR, Winterford CM, Harmon BV (1994). Apoptosis: its significance in cancer and cancer therapy. *Cancer*.

[9] Malhi H, Guicciardi ME, Gores GJ (2010). Hepatocyte death: a clear and present danger. *Physiological Reviews*.

[10] Gavrieli Y, Sherman Y, Ben-Sasson SA (1992). Identification of programmed cell death in situ via specific labeling of nuclear DNA fragmentation. *Journal of Cell Biology*.

[11] Takemura G, Kato S, Aoyama T (2001). Characterization of ultrastructure and its relation with DNA fragmentation in Fas induced apoptosis of cultured cardiac myocytes. *The Journal of Pathology*.

[12] Huerta S, Goulet EJ, Huerta-Yepez S, Livingston EH (2007). Screening and Detection of Apoptosis. *Journal of Surgical Research*.

[13] Yin F, Giuliano AE, van Herle AJ (1999). Signal pathways involved in apigenin inhibition of growth and induction of apoptosis of human anaplastic thyroid cancer cells (ARO). *Anticancer Research*.

[14] Hirano T, Higa S, Arimitsu J (2004). Flavonoids such as luteolin, fisetin and apigenin are inhibitors of interleukin-4 and interleukin-13 production by activated human basophils. *International Archives of Allergy and Immunology*.

[15] Nakazawa T, Yasuda T, Ueda J, Ohsawa K (2003). Antidepressant-like effects of apigenin and 2,4,5-trimethoxycinnamic acid from Perilla frutescens in the forced swimming test. *Biological and Pharmaceutical Bulletin*.

[16] Safari M-R, Sheikh N (2003). Effects of flavonoids on the susceptibility of low-density lipoprotein to oxidative modification. *Prostaglandins, Leukotrienes and Essential Fatty Acids*.

[17] Begum N, Prasad NR, Thayalan K (2012). Apigenin protects gamma-radiation induced oxidative stress, hematological changes and animal survival in whole body irradiated Swiss albino mice. *International Journal of Nutrition, Pharmacology, Neurological Diseases*.

[18] Huguet C, Addario-Chieco P, Gavelli A, Arrigo E, Harb J, Clement RR (1992). Technique of hepatic vascular exclusion for extensive liver resection. *The American Journal of Surgery*.

[19] Delva E, Camus Y, Nordlinger B (1989). Vascular occlusions for liver resections. Operative management and tolerance to hepatic ischemia: 142 cases. *Annals of Surgery*.

[20] Henderson JM (1999). Liver transplantation and rejection: an overview. *Hepato-Gastroenterology*.

[21] Zweier JL, Talukder MAH (2006). The role of oxidants and free radicals in reperfusion injury. *Cardiovascular Research*.

[22] Lemasters JJ (1999). V. Necrapoptosis and the mitochondrial permeability transition: shared pathways to necrosis and apoptosis. *The American Journal of Physiology*.

[23] Hirsch T, Marchetti P, Susin SA (1997). The apoptosis-necrosis paradox. Apoptogenic proteases activated after mitochondrial permeability transition determine the mode of cell death. *Oncogene*.

[24] Elmore S (2007). Apoptosis: a review of programmed cell death. *Toxicologic Pathology*.

[25] Malhi H, Gores GJ, Lemasters JJ (2006). Apoptosis and necrosis in the liver: a tale of two deaths?. *Hepatology*.

